# 制备两种p53重组腺病毒和流式细胞仪定量外源绿荧光蛋白表达

**DOI:** 10.3779/j.issn.1009-3419.2010.05.17

**Published:** 2010-05-20

**Authors:** 惠 汪, 百塘 赖, 伟英 李, 学惠 杨, 春燕 张, 攀健 韦, 金照 李

**Affiliations:** 1 101149 北京，北京市结核病胸部肿瘤研究所 Department of Cell-Molecular Biology, Beijing TB and Toracic Tumor Research Institute, 101149 Beijing, China; 2 100101 北京，中国科学院生物物理所 Institute of Biophysics, Chinese Academy of Science, 100101 Beijing, China

**Keywords:** 野生型p53, 缺失C-末端p53, 3’端非编码区, 重组腺病毒, 流式细胞仪散点图, 绿荧光蛋白表达, Wild type p53, Deletion C-terminal p53, 3'Untranslation region, Recombinant defcient adenoviruses, Flow cytometry scater plot, Expression of green fluorescence protein

## Abstract

**背景与目的:**

p53作为转录因子，在细胞应激时呈活化型，可调控细胞周期和程序性死亡抑制肿瘤生长，通常通过各种机制可使p53呈现非活化状态，其中包括p53 C-末端负调控序列的作用。本研究旨在制备携带全长和缺失这些负调控序列p53的两种重组腺病毒，并采用流式细胞仪散点图（flow cytometry scater plot, FCM）检测人肺癌细胞外源绿荧光蛋白（green fluorescence protein, GFP）表达。

**方法:**

利用pAdEasy-Track载体系统，构建两种p53重组质粒并在细菌中产生重组体，转染L293细胞产生三种重组腺病毒，测序证明。三种不同浓度病毒分别感染人肺癌801D细胞，FCM scater plot检测其GFP表达。

**结果:**

测序证明重组腺病毒：Ad-p53(del)缺失p53 C-末端终止密码子前111个碱基和非编码区，Ad-p53(wtp)无p53碱基缺失。Ad-(empty carrier)无p53。FCM scater plot显示三种病毒感染801D细胞表达GFP百分率接近并随病毒浓度递增。801D包含了不同荧光强度比率的细胞。

**结论:**

构建和制备了去C-末端p53和全长p53的两种重组腺病毒：Ad-p53(del)、Ad-p53(wtp)及空载体Ad-(empty carrier)。流式细胞仪散点图证明该病毒试验系统可靠，可定量外源GFP表达为病毒感染细胞选择浓度提供准确方法。

## Abstract (en)

**Background and objective:**

The p53 as a transcription factor in cell stress was activated to regulate cell cycle and programmed cell death to inhibit tumor growth. Usually, p53 is kept in non-activated state through various mechanisms, including the action of p53 C-terminal negative regulatory sequences. The purpose of the study is to prepare the two types p53 recombinant adenoviruses that carry full-length p53 as well as deletion of negative regulatory sequences at p53 C-terminus and to detect exogenous GFP expression in human lung cancer cell infected-virus by FCM scater plot.

**Methods:**

Using pAdEasy-Track vector system the p53 recombinant plasmids was constructed and the homologous recombinants in *E.coli* was produced. The three kinds of recombinant adenovirus in L293 cells was generated, sequencing proved. Exogenous GFP expression in human lung cancer 801D cells infectedvirus was detected by FCM scater plot.

**Results:**

p53 recombinant adenoviruses named Ad-p53(wtp), Ad-p53(del) and Ad-(empty carrier) were produced. Results of sequences indicate that the Ad-p53(del) was deletion of 111 bases before stop codon TGA and of 3 untranslated region at p53, the Ad-p53(wtp) no loss of any p53 base, the Ad-(empty carrier) no p53 sequence. FCM scater plot indicate the percentage of 801D cells expressed GFP with three kinds of viral infection was almost same and was increased with the virus density. 801D contains ratio of cells with different?uorescence intensity.

**Conclusion:**

The preparation of recombinant adenovirus, Ad-p53(del), pA-p53(wtp) and Ad-(empty carrier). The cells expressed-GFP can be quantitatively detected by FCM scater plot. It was provide that the reliability of the virus system and accurate method for selecting viruses density to infecting cells.

我们在体外研究中发现去除C-末端负调控区的p53比全长p53在体外抑制人肺癌细胞的增殖作用更强^[1]^。为了进一步深人研究去除p53 C-末端的生物学意义，该研究采用5型缺陷型腺病毒pAdEasy-Track载体系统^[2]^，构建和制备了去C-末端p53和全长p53重组腺病毒，命名为Adp53(del)和Ad-p53(wtp)。质粒Track上有2个CMV启动子，可分别启动下游绿荧光蛋白（green fluorescence protein, GFP）基因和重组基因表达。GFP在细胞中表达可作为病毒感染和转导外源基因效果的标记。通常，用荧光显微镜检测细胞GFP表达，但方法不够准确。该研究用流式细胞仪散点图（flow cytometry scatter plot, FCM）可量化表达GFP细胞的百分率，为确定该病毒试验系统的可靠性和病毒感染人肺癌细胞浓度提供准确方法。

## 1 材料与方法

### 1.1 细胞系

人大细胞肺癌细胞系801D，由中国人民解放军301医院提供。

### 1.2 缺陷型重组腺病毒的制备

按Tong-Chuan He^[2]^报告pAdEasy-Track载体系统和方法构建。

#### 1.2.1 构建两种类型p53重组质粒

Track-p53(del)和Trackp53(wtp) pAdEasy-N1（5型缺陷型腺病毒骨架）和Track由北京肿瘤研究所张志谦教授提供。用*Bam*H1酶切重组质粒pEGFP-N1-p53(del) cDNA和PEGFP-N1-p53(wtp) cDNA获去C-末端p53和全长p53片段。经质粒Track多克隆位点*Big*Ⅱ（*Bam*H1同位酶）插入构建重组质粒，转染大肠菌*E.coli*，卡那霉素筛选耐受菌落。提DNA经*Awl*nl和*Hin*di双酶切重组质粒，酶切图篩选。

#### 1.2.2 在细菌中产生同源重组体

pAdT-Ep53(del)、pAdT-Ep53(wtp)、pAdT-E。用酶*pac*1消化Track、Trackp53(del)、Track-p53(wtp)成线性，分别与*pac1*消化的pAdEasy-N1混合，用电转移方法转染大肠感受态杆菌（*E.coli* BJ5183株），Track和pAdEasy-N1在细菌中同源重组，kanamycin筛选耐受菌，挑选琼脂培养基上的集落扩增，提取DNA，经琼脂电泳分析DNA，选择超螺旋构象重组体，并进一步用*pac*1酶切图证明。经*Xho*1、*Eco*R1酶切图筛选三种重组体。

#### 1.2.3 在293包装细胞中制备缺陷型重组腺病毒

Adp53(del)、Ad-p53(wtp)、Ad-(empty carrier)。接种293细胞（1.5×10^5^/3 cm平皿），lipofectamin分别转染三种同源重组体，荧光显微镜监测细胞中GFP表达。7 d-10 d收集细胞和上清，冻存，制备缺陷型腺病毒。

#### 1.2.4 重组质粒、同源重组体、重组腺病毒DNA测序

重组质粒：Track-p53(wtp)、Track-p53(del)，同源重组体：AdT-Ep53(del)、pAdT-Ep53(wtp)、pAdT-E，重组腺病毒；Ad-p53(del)、Ad-p53(wtp)、Ad-(empty carrier)提DNA由上海生工测序，经Gene Bank公共数据库比对（blast）。

#### 1.2.5 病毒浓度（multiplicity of infection）测定^[2]^

接种293包装细胞（5×10^6^/3 cm/平皿），取病毒原液稀释10倍后分别取0.75 μL、1.5 μL、3 μL、6 μL、12.5 μL、25 μL感染细胞，3 d后，FCM检测801D细胞GFP表达。按下列公式计算每微升原液病毒的数量。

$
{\rm{每微升原液病毒的数量 = }}\frac{{{\rm{接种}}293{\rm{细胞数}} \times {\rm{10}}}}{{\rm{病毒原液最小体积}}}
$

病毒原液最小体积是指上述稀释病毒液感染细胞平皿中90%以上293细胞表达GFP所加入最少的病毒体积。接种293细胞数×10是指接种的细胞中每个细胞约含有10倍病毒。

#### 1.2.6 病毒感染人肺癌细胞在裸鼠体内表达

接种2×10^6^ 801D细胞，分别用于细胞50倍的三种重组腺病毒感染细胞接种裸鼠皮下，24 h后，取接种部位细胞团压片，于荧光显微镜检测细胞GFP。

### 1.3 荧光显微镜和流式细胞仪检测体外感染细胞GFP表达

#### 1.3.1

病毒感染801D细胞：制备3×10^5^ 801D细胞悬液，稀释10倍后，分别用细胞的25、50、100、200倍3种病毒感染细胞，3 h后接种于平皿（3 cm）。37 ℃、CO_2_暖箱培养24 h。行荧光显微镜检测，同时消化，洗涤，稀释细胞，行FCM检测细胞。

#### 1.3.2

荧光显微镜（激发光为488 nm，发射光波长为507 nm滤光片）检测细胞GFP表达。

#### 1.3.3

FCM scatter plot（激发光为488 nm）检测细胞GFP表达。R6为无GFP表达细胞比率，R2、R3、R4、R5为表达GFP细胞和不同GFP荧光强度细胞的百分率（从弱到强）。

## 2 结果

### 2.1 去除C未端p53和全长p53重组质粒的构建

构建去C-末端p53和全长p53重组质粒Track-p53(del)和Trackp53(wtp)，经*Awl*nl和*Hin*d酶切图筛选。

### 2.2 重组质粒与pAdEasy-N1在细菌中产生同源重组体

DNA琼脂电泳构像不同，选择重组体（图 1）。由于Track上有两个*pac*1酶切点，Easy上有一个酶切点，用*pac*1酶切重组体，可产生一个30 kb大片段和约3 kb-4 kb小片段（图 2）。经*Xho*1、*Eco*R1酶切图筛选Track-Eazyp53(del)、Track-Easy-p53(wtp)和Track-Easy（图 3）。

**1:**
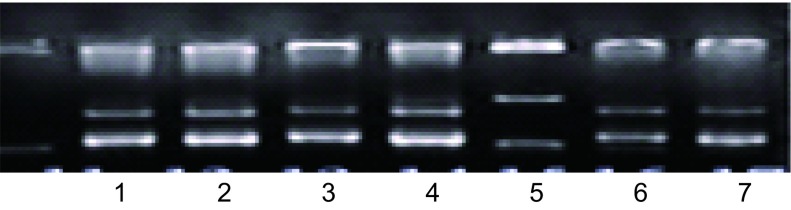
不同集落DNA在琼脂电泳上构象选择同源重组体。5号为超螺旋同源重组体。 DNA conformation of different clone on agarose gel electroporator to select homologous recombinants. The number 5 is homologous recombinant of supercoiled form.

**2:**
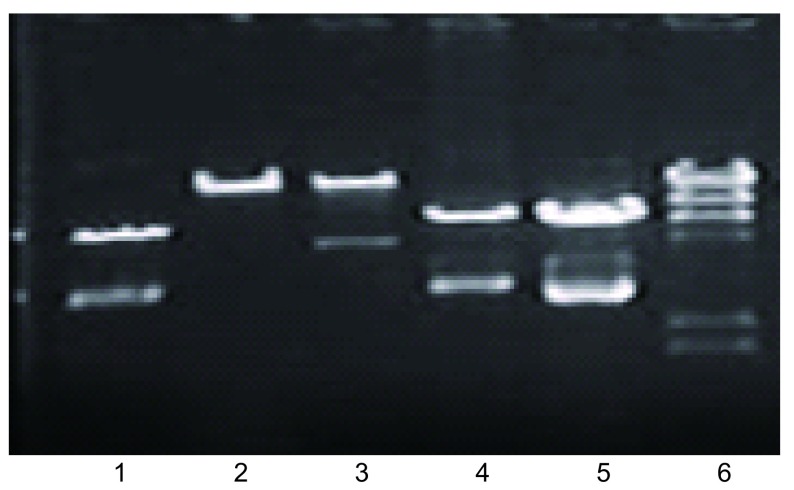
*Pac*1酶切图证明重组体 *Pac* 1 restrictive endonuclease map of two p53 recombinants. 1: Track; 2: Easy; 3: T-E-p53(wtp); 4: 5.T-E-p53(del); 6: marker(*λ*-*Hin* dIII).

**3:**
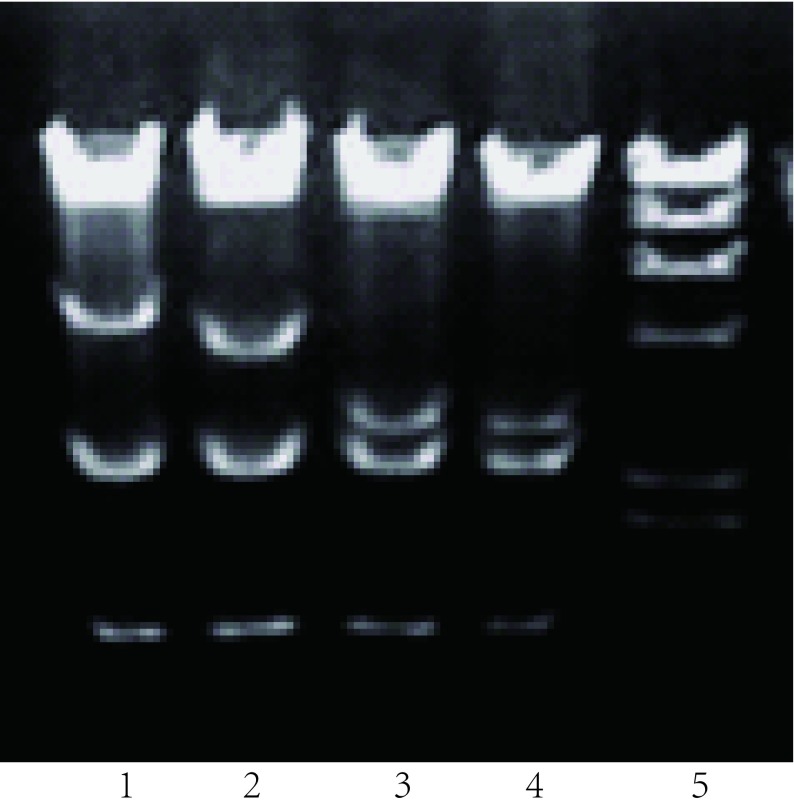
*Xho* 1、*Hin* dⅢ酶切图证明两种重组p53 *Xho* 1, *Hin*dⅢ restrictive endonuclease map of two p53 recombinants. 1: T-Ep53(wtp); 2: T-Ep53(del); 3, 4: T-E; 5: Marker(*λ*-*Hin*dⅢ).

### 2.3 三种重组腺病毒制备

在L293细胞中制备了三种重组腺病毒：Ad-(empty carrier)、Ad-p53(del)和Adp53(wtp)。可见细胞中GFP表达（图 4）。

**4:**
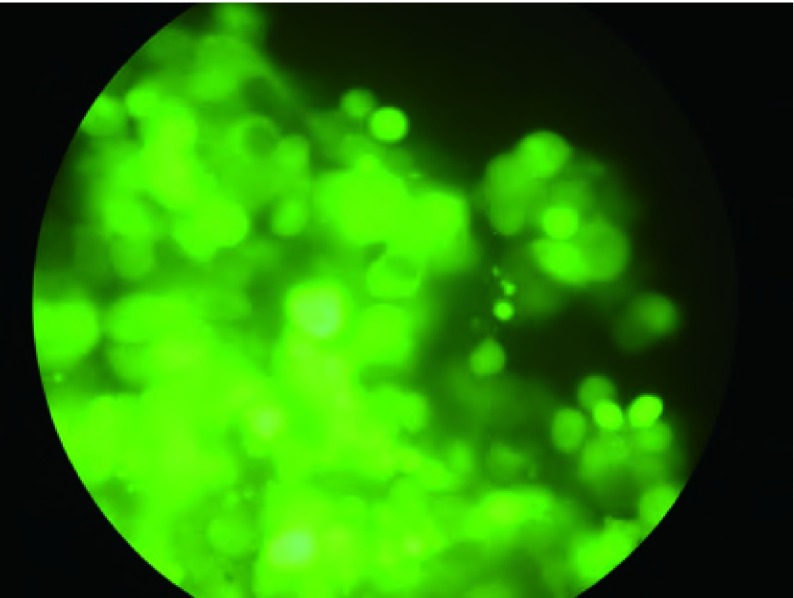
L293细胞系中产生重组腺病毒 Generation of pAdEasy-p53(del) in L293 cell line

### 2.4 测序结果（图 5）

两种重组质粒测序，经Gene Bank公共数据库blast证明Track-p53(wtp)无碱基缺失（图 5A）。Track-p53(del)在p53终止密码子TGA前有111个碱基缺失和终止密码子后的所有非编码序列缺失（图 5B）。三种同源重组体（图 5C、D、E）和三种p53重组腺病毒（图 5F、G、H）测序结果经blast证明p53序列与重组质粒Track-p53(del)和Track-p53(wtp)结果相同。Track-Easy、Ad-(empty carrier)无p53序列。

**5:**
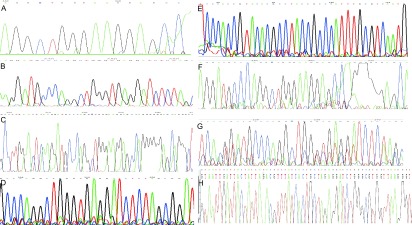
重组质粒, 重组体和组腺病毒测序结果。A：Track-p53(wtp) DNA，C：T-E p53(wtp) DNA，F: Ad-p53(wtp) DNA，测序结果均无p53碱基缺失；B：Track-p53(del) DNA，D：T-E p53(del) DNA，G：Ad-p53(del) DNA，测序结果终止密码子前111个碱基和终止密码子后所有非编码序列缺失；E：T-EDNA sequence，H：Ad-(empty carrier) DNA测序结果无p53序列。 The sequence of the recombinant plasmids, the homologous recombinants and the recombinant adenovirus. A: Track-p53(wtp) DNA, C: T-E p53(wtp) DNA, F: Ad-p53(wtp) DNA. Without deletion any bases at p53; B: Track-p53(del) DNA, D: T-E p53(del) DNA, G: Ad-p53(del) DNA. Deletion 111 bases before stop codon TGA and 3' untranslated region after stop codon TGA at p53; E: T-E DNA sequence, H: Ad-(empty carrier) DNA. Without any p53 sequence.

### 2.5 病毒浓度

FCM scatter plot显示90%以上L293细胞表达GFP，最小病毒感染细胞浓度：Ad-(empty carrier)、Adp53(wtp)为1.5 μL和Ad-p53(del)为3 μL（表 1）。按上述M0i公式计算A、C病毒浓度为3.7×10^10^/mL，B病毒为1.6×10^10^/mL。

**1:** 流式细胞仪检测L293细胞GFP表达 Detection GFP expression on L293 cells by FCM

Viruses	Concentration of viruses										
	0.7 µL			1.5 µL			3 µL			6 µL	
	Cells (%)			Cells (%)			Cells (%)			Cells (%)	
	GFP (-)	GFP (+)		GFP (-)	GFP (+)		GFP (-)	GFP (+)		GFP (-)	GFP (+)
Infectingng cells with Ad-p53(wtp)	14	86		7	93		10	90		7	93
Infection cells with Ad-p53(del)	36	64		13	87		6	94		6	94
Infection cells with Ad-(empty carrier)	18	82		5	95		5	95		6	94
Without infecting	99.7	0.3									

### 2.6 三种重组腺病毒感染人肺癌801D细胞GFP表达结果

#### 2.6.1

病毒感染的801D细胞接种裸鼠体内24 h后细胞GFP开始表达（图 6）。

**6:**
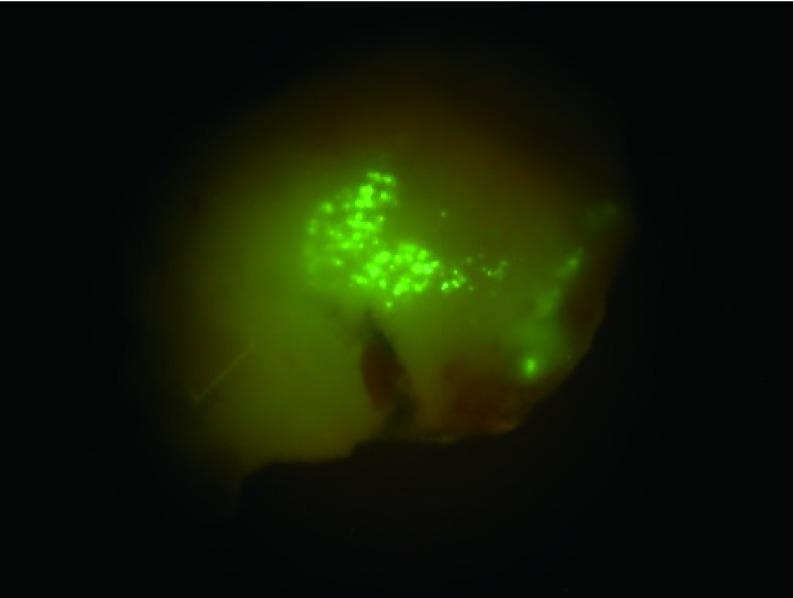
转染Ad-p53(del)病毒的细胞在裸鼠体内表达GFP GFP expression of cells infected by Ad-p53(del) *in vivo*

#### 2.6.2 荧光显微镜下观察801D GFP表达

25、50、100、200倍三种病毒感染的801D细胞在荧光显微镜下观察到细胞GFP表达（图 7）。对照细胞（无感染病毒的801D）无GFP表达。感染的801D细胞随病毒浓度增加，表达GFP细胞的数量增加。细胞中有不同荧光强度细胞，随病毒浓度增加，细胞荧光强度增加。同一浓度不同种类病毒GFP表达结果相近。但不能准确估计各病毒浓度对细胞的感染率。

**7:**
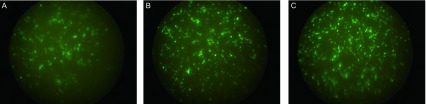
荧光显微镜检测不同浓度Ad-p53(del)病毒感染801D细胞GFP表达。50x（A），100x（B），200x（C）：转染细胞的病毒浓度。 Detection GFP expression by fluorescent microscopy in 801D cells infected with different density Ad-p53(del). 50x (A), 100x (B), 200x (C): different virus density to infect cells.

#### 2.6.3 FCM scatter plot检测结果

结果显示对照细胞（无感染病毒的801D）99.9%无GFP表达（R6）（图 8D）。三种病毒感染的细胞均表达GFP（R2-R5）（图 8A、B、C；表 2）。随病毒浓度增加，细胞GFP表达率增加。Ad-p53(wtp)病毒感染（图 8A）：25倍有47%细胞表达GFP。50倍有68%。100倍有79%。200倍有95%。Adp53(del)病毒（图 8B）：25倍有37%细胞表达GFP，50倍有71%，100倍有74%，200倍为90%。Ad-(empty carrier)病毒感染801D（图 8C）：25倍感染有46%细胞表达GFP，50倍有62%，100倍有79%，200倍有94%。同一浓度不同病毒感染细胞表达GFP细胞的比率相近。此外，FCM scatter plot还显示了三种病毒感染的801D细胞群体中包含了表达不同荧光强度（R2、R3、R4、R5）细胞的百分率（表 3，图 9）。表达低荧光强度（R2）细胞约占9%，不随病毒浓度增加，主要分布在低浓度感染的细胞。表达中等荧光强度（R3）的细胞比率最高，约占28%。细胞比率不随病毒浓度增加而改变。高荧光强度（R4、R5）细胞分别约占20%、13%，主要分布在高浓度病毒（100倍、200倍）感染的细胞。提示801D由表达不同GFP强度的细胞组成。

**2:** 流式散点图检测不同病毒感染801D细胞表达GFP百分率 Detection of percentage of 801D cell GFP-expressed by FCM scatter plot

Group	Express GFP cells (%)				
	0	25x	50x	100x	200x
Ad-p53(wtp)					
viruses infecting 801D					
GFP (-) cells (R6)		53	32	11	5
GFP (+) cells		47	68	89	95
Ad-p53(del)				
viruses infecting 801D					
GFP (-) cells (R6)		63	43	26	10
GFP (+) cells		37	57	74	90
Ad-(empty carrier)				
viruses infecting 801D					
GFP (-) cell (R6)		54	38	21	7
GFP (+) cell		46	62	79	93
Without infecting 801D					
GFP (-) cell (R6)		99.9			
GFP (+) cell		0.1			

**8:**
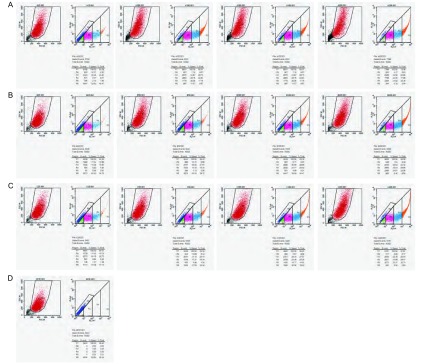
FCM scatter plot检测801D细胞GFP表达。A：Ad-p53(wtp)转染801D细胞GFP表达；B：Ad-p53(del)转染801D细胞GFP表达；C：Ad-(empty carrier)转染801D细胞GFP表达；D：无转染801D。 Detection GFP expression in cells infected-viruses by FCM scatter plot. A: GFP expression in cells infected-Ad-p53(wtp); B: GFP expression in cells infected-Ad-p53(del); C: GFP expression in cells infected-Ad-(empty carrier); D: Without GFP expression in control 801D cells.

**3:** 流式散点图显示表达不同荧光强度细胞分布 FCM scatter plot detection of distribution of fluorescent intensity in 801D cells infected with different viruses

Different viruses	Cell prcentage of different fluorescence intensity				
	R2	R3	R4	R5	Total
Ad-p53(wtp)					
25x	10	26	10	2	48
50x	10	34	19	7	70
100x	7	35	27	21	90
200x	4	35	24	32	95
Ad-p53(del)					
25x	12	19	6	0.6	38
50x	11	30	14	3	58
100x	10	29	25	16	80
200x	6	22	32	33	93
Ad-(empty carrier)					
25x	11	25	10	1	47
50x	10	31	17	5	63
100x	10	31	24	10	75
200x	7	27	33	25	92
R2→R5: fluorescent intensity from low to high.					

**9:**
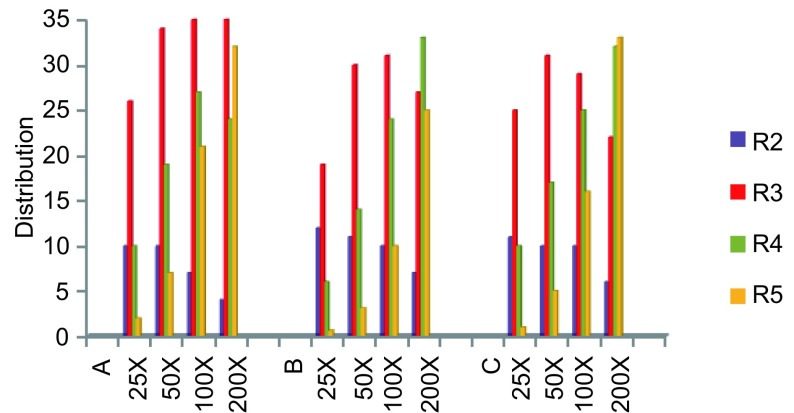
流式显示表达不同荧光强度细胞的分布 FCM scatter plot indicate GFP distribution to cells infected by Adp53(wtp) (A), Ad-p53(del) (B) and Ad-(empty carrier) (C)

## 3 讨论

抑癌基因*p53*作为转录因子在细胞应激时呈活化状态，通过调节细胞增殖周期和细胞程序性死亡抑制肿瘤生长，通常处于非活化型，通过各种机制包括p53 C-末端负调控区的作用所致^[3-5]^。先前，我们利用转基因细胞在体外试验中已证明了外源去C-末端p53比全长p53抑制人肺癌细胞801D的增殖作用更强。并发现可增加对抗肿瘤药物敏感性（尚未发表）。重组腺病毒是研究外源基因表达及生物学功能和基因治疗的重要载体^[6]^。该文报告了采用pAdEasy-Track载体系统构建和制备了去C-末端p53和全长p53两种重组腺病毒Ad-p53(del)、Ad-p53(wtp)以及空载体Ad-(empty carrier)。该载体系统可快速大量产生重组腺病毒为进一步深人研究p53C-末端负调节功能提供了条件。病毒转导外源基因的效果是进行研究的必要前提。由于病毒、细胞、感染方法和实验条件等诸多因素可影响到病毒载体对细胞感染和转基因效果，因此建立精确检测转导基因效果的方法用于估价所建立的病毒试验系统的可靠性和准确性是重要的。通常，通过重组质粒上的标记基因表达作为病毒感染细胞和转基因的标记。该研究所用质粒Ad Track上有2个CMV启动子，分别起动GFP标记基因和外源基因表达。通常用荧光显微镜检测细胞GFP表达，但结果不够精确。用FCM检测可精确显示GFP表达^[7, 8]^。本研究采用FCM检测L293细胞GFP表达可精确确定重组腺病毒的浓度。在分别用25、50、100、200倍重组腺病毒感染人肺癌801D细胞后，用荧光显微镜和FCM两种方法检测细胞。结果显示荧光显微镜观测可见表达GFP细胞随病毒浓度递增，三种病毒感染细胞表达GFP效果比较接近。并可见表达GFP细胞群体中包含了表达不同荧光强度的细胞。但不能准确估计表达GFP细胞比率。FCM scatter plot能够准确显示感染细胞GFP表达百分率。随病毒浓度递增。不同病毒相同浓度感染，细胞GFP表达百分率接近。结果为选择病毒感染细胞的合适浓度做了提示。此外，scatter plot显示801D细胞群体中包含了4种不同荧光强度比率的细胞。表达低荧光强度（R2）细胞约占9%，不随病毒浓度增加。表达中等荧光强度（R3）的细胞比率最高，约占28%，病毒浓度增加，细胞比率无明显改变。高荧光强度（R4、R5）细胞分别约占20%、13%，主要分布在高浓度病毒（100倍、200倍）感染的细胞。探其缘由可能与肿瘤细胞表面的病毒特异性科萨奇腺病毒受体（coxsackie and adenovirus receptor, CAR）以及整合素（integrin）的表达水平不同有关。在腺病毒对肿瘤细胞的感染和转导外源基因表达中它们起着决定性作用^[9]^。801D存在表达特异性表面受体和整合素水平不同的细胞是肿瘤细胞异质性的表现^[10]^。该结果提示针对不同肿瘤患者采用不同浓度的重组腺病毒进行基因治疗可能更有利于患者治疗。总之，FCM scatter plot检测所显示的优点是重组腺病毒载体转导外源基因生物学实验和重组腺病毒为载体的临床基因治疗中值得推崇的方法。

本研究采用pAdEasy-Track载体系统成功构建和制备了去C-末端p53和全长p53两种重组腺病毒：Adp53(del)、Ad-p53(wtp)以及空载体Ad-(empty carrier)。FCM scatter plot定量显示感染细胞中表达外源GFP细胞百分率和表达不同荧光强度细胞的百分率，证明了该病毒试验系统可靠性和为选择病毒感染人肺癌细胞801D的合适浓度提供准确方法。
